# Supplementary material for: In vivo Imaging of Cannabinoid Type 2 Receptors: Functional and Structural Alterations in Mouse Model of Cerebral Ischemia by PET and MRI
Source: Mol Imaging Biol. 2021 Oct 12;24(5):700–9. doi: 10.1007/s11307-021-01655-4 (PMC9581861; doi:10.1007/s11307-021-01655-4)
Supplement: Supplementary file 1 — Supplementary file1 (DOCX 1129 kb) [file 11307_2021_1655_MOESM1_ESM.docx]

**Supplementary information:**

**Supplementary Figure 1.** Time activity curves of [^18^F]RoSMA-18-d6 *in vivo* microPET imaging in tMCAO mouse brain. (**a-d**) In the cortex, striatum, cerebellum and midbrain under baseline (n = 6) and blockade (n = 4) conditions. No difference in [^18^F]RoSMA-18-d6 SUV was observed in different brain regions at ipsilateral vs contralateral side under baseline or blockade conditions. Data represent mean ± standard deviation.


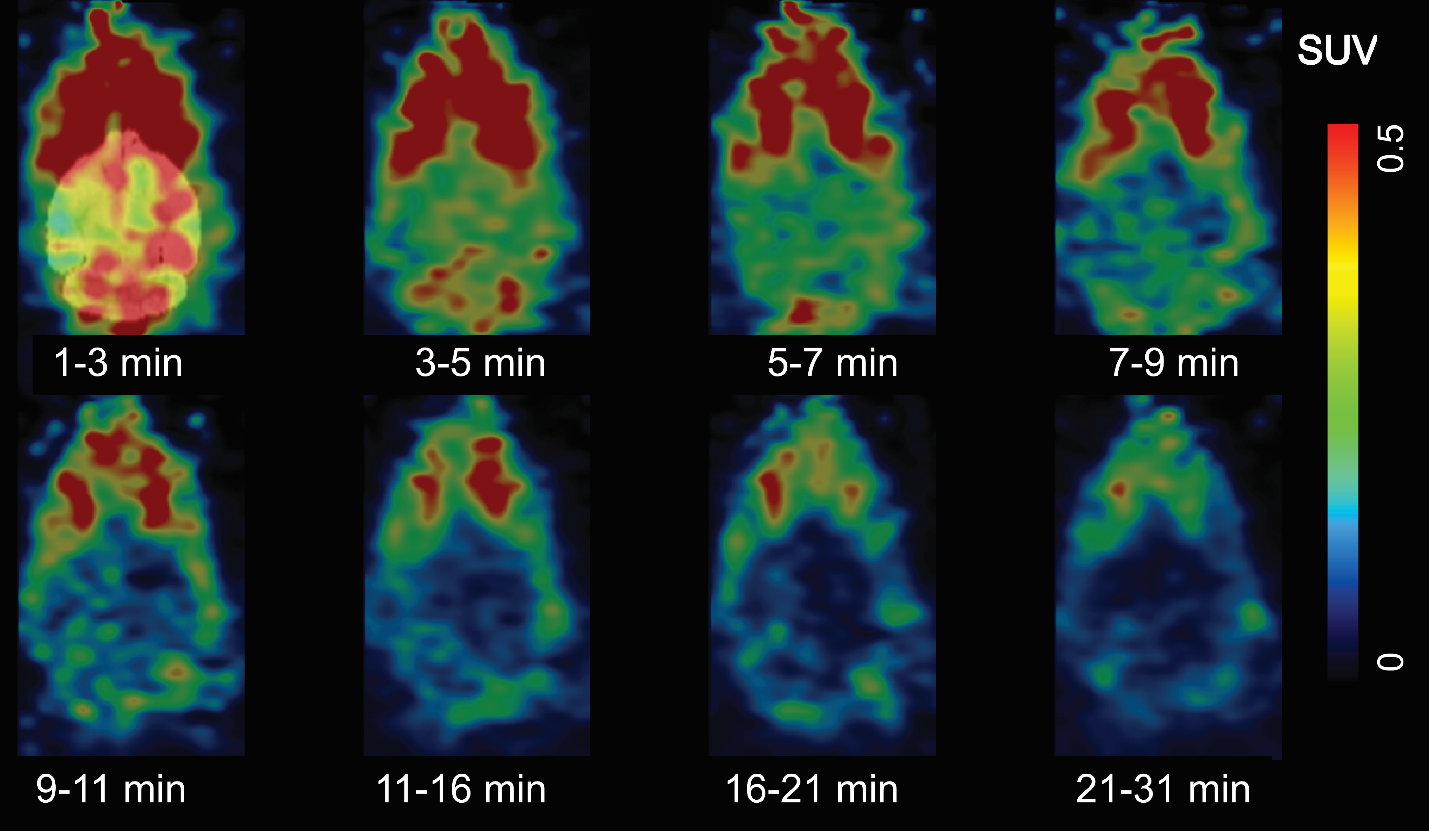


**Supplementary Fig 2.** *In vivo* microPET imaging of tMCAO mouse brain using [^18^F]RoSMA-18-d6. Representative whole head PET images of horizontal mouse brain sections at different time frames after intravenous injection of [^18^F]RoSMA-18-d6; SUV: 0-0.5;

**Supplementary Table 1: Primers used for the quantitative polymerase chain reaction assay on mouse brain tissue**

| RNA | Primer |
| --- | --- |
| *beta-actin (ACTB)* | forward 5′-AGACCTCTATGCCAACACAGT-3′, reverse 5′-TGCTAGGAGCCAGAGCAGTAA-3′ |
| *Cannabinoid Receptor 2 (CNR2)* | forward 5′-CTACAAAGCTCTAGTCACCCGT-3′, reverse 5′-CCATGAGCGGCAGGTAAGAAA-3’ |
| *ionized calcium binding adaptor molecule 1 (Iba1)* | forward 5’-GTCCTTGAAGCGAATGCTGG-3’, reverse 5’-CATTCTCAAGATGGCAGATC-3’ |
| *Tumor necrosis factor (TNF-a)* | forward 5′-AATGGCCTCCCTCTCATCAGTT-3′, reverse 5′-CCACTTGGTGGTTTGCTACGA-3′ |
| *Matrix metallopeptidase 9 (MMP9)* | forward 5’-AACATCTGGCACTCCACACC-3’, reverse 5’-GCAGAAGTTCTTTGGCCTGC-3’ |
| *Glial fibrillary acidic protein (GFAP)* | forward 5’-CGGAGACGCATCACCTCTG-3’, reverse 5’-TGGAGGAGTCATTCGAGACAA-3’ |
| *microtubule-associated protein 2 (MAP-2)* | forward 5’-GCCAGCCTCAGAACAAACAG-3’, reverse 5’-AAGGTCTTGGGAGGGAAGAAC-3’ |
